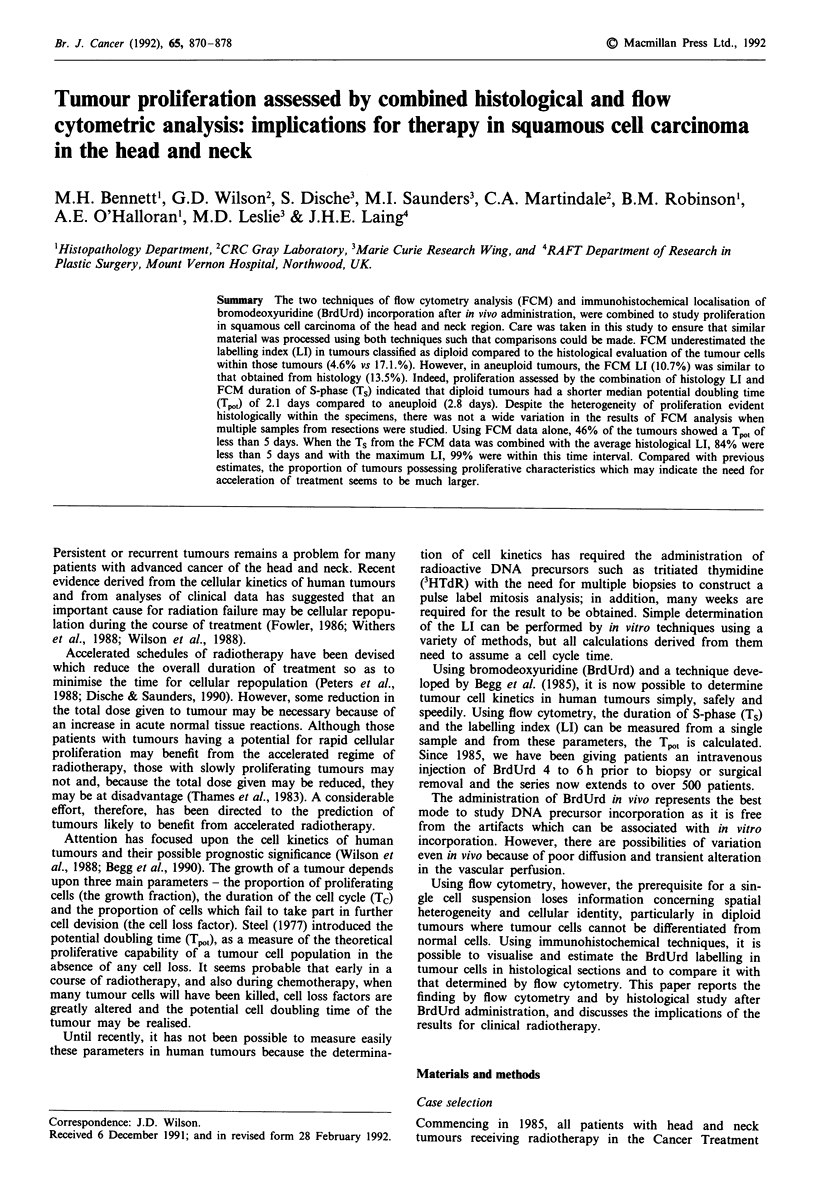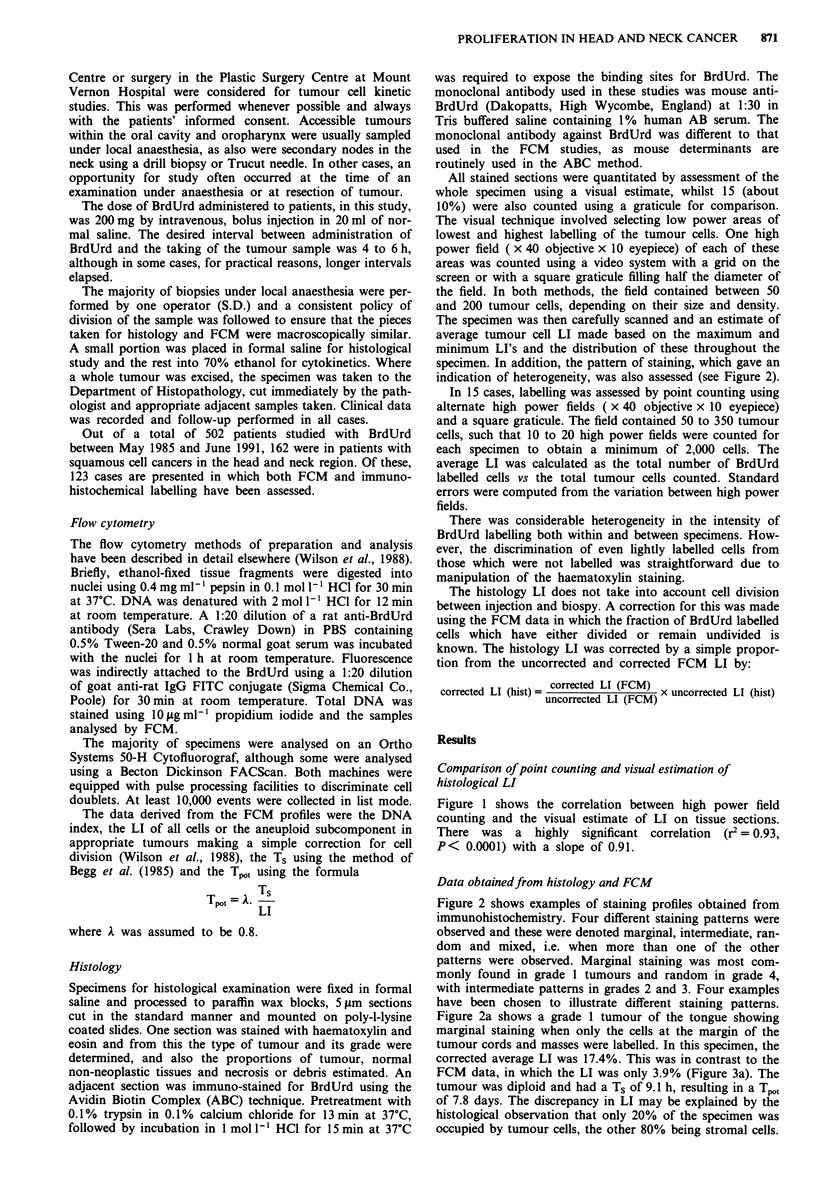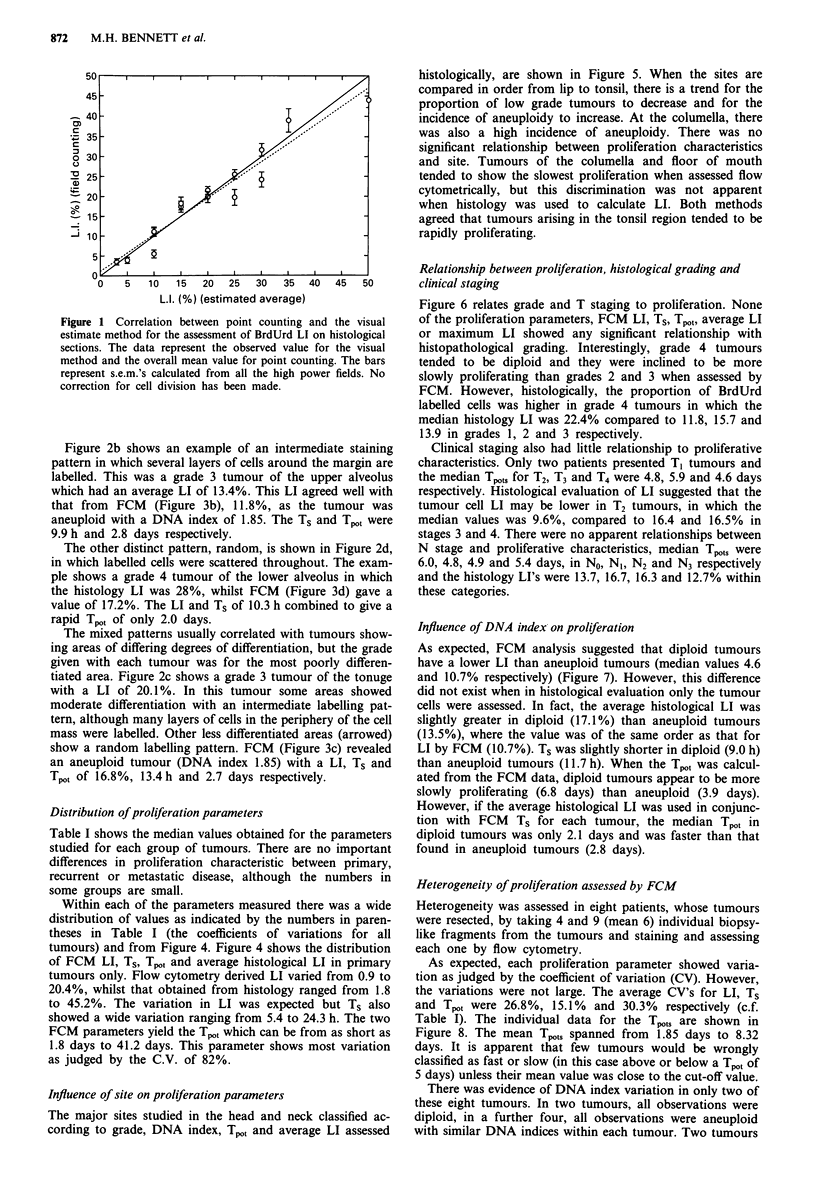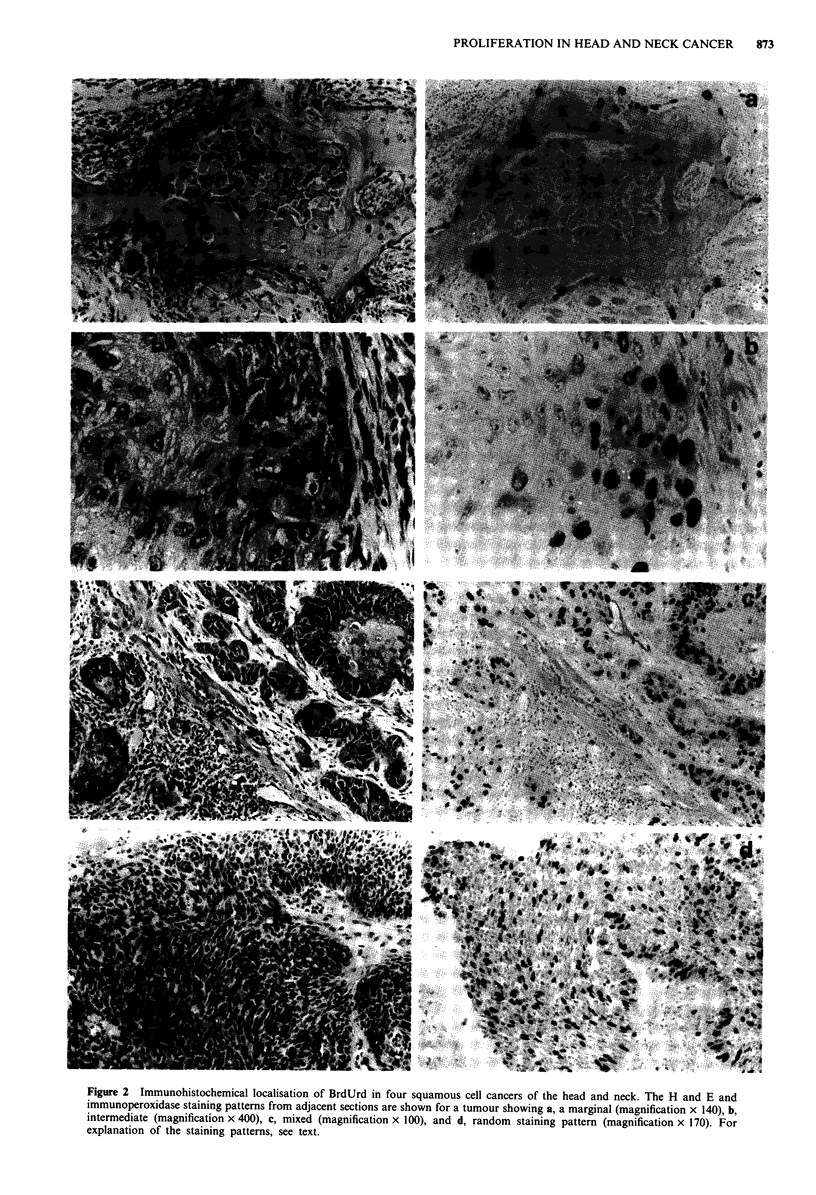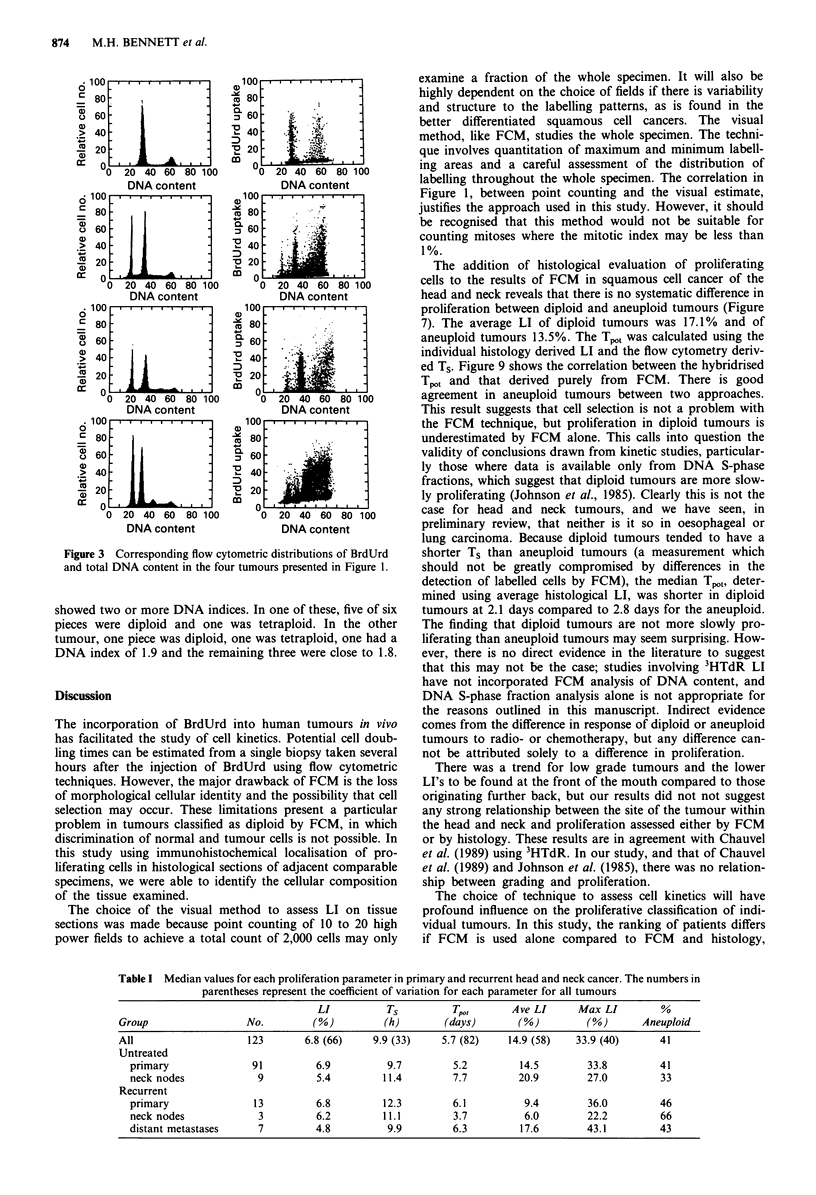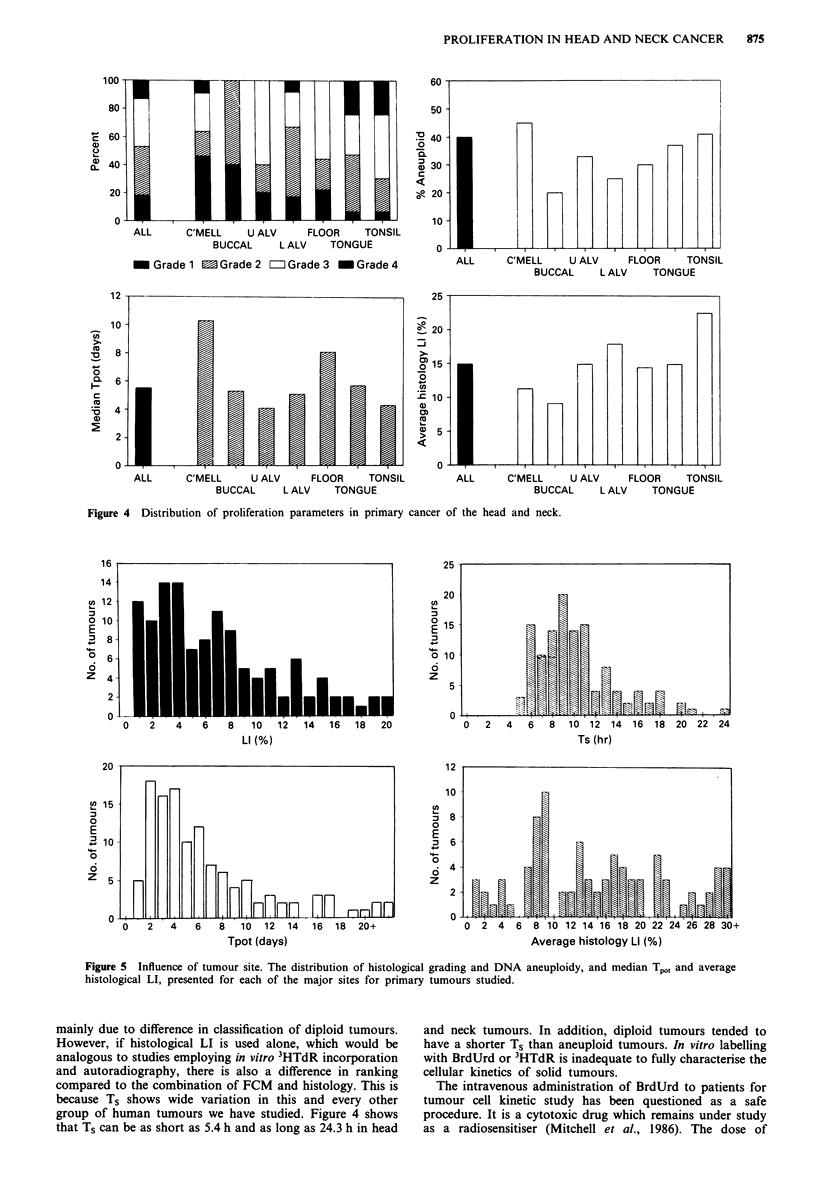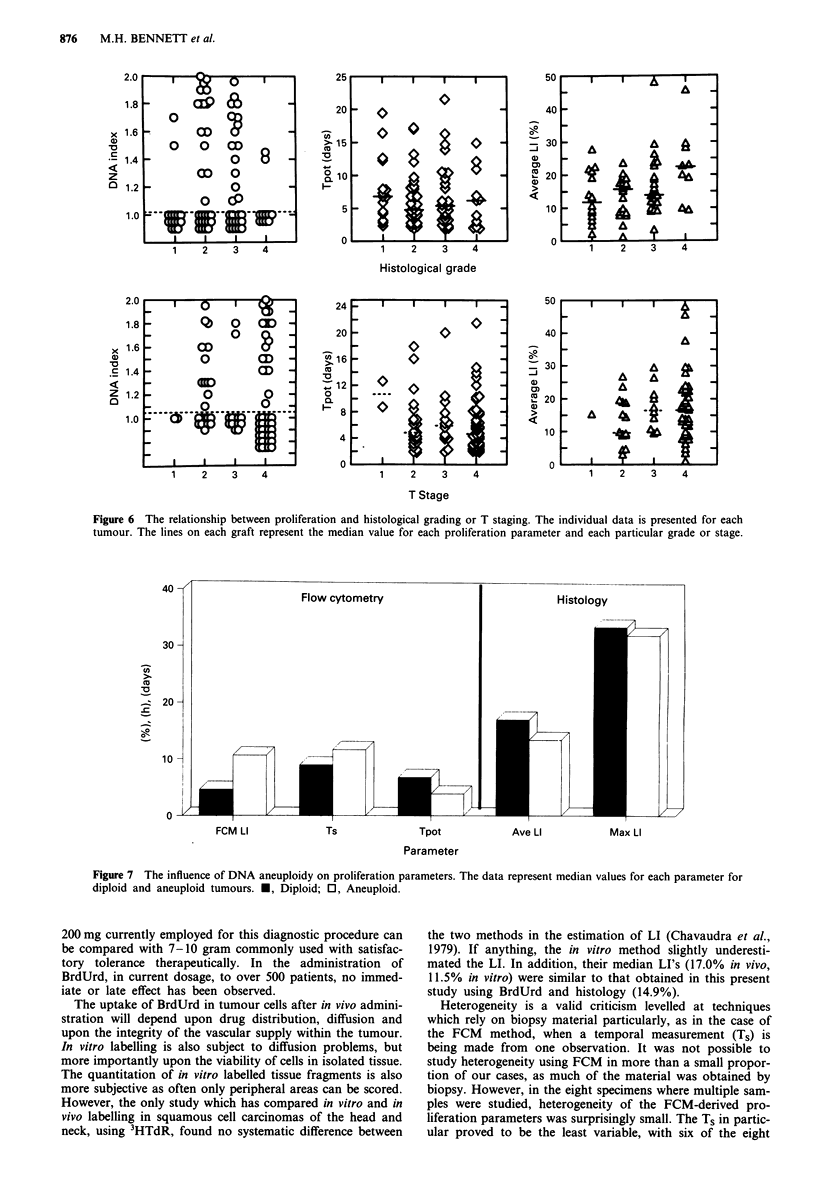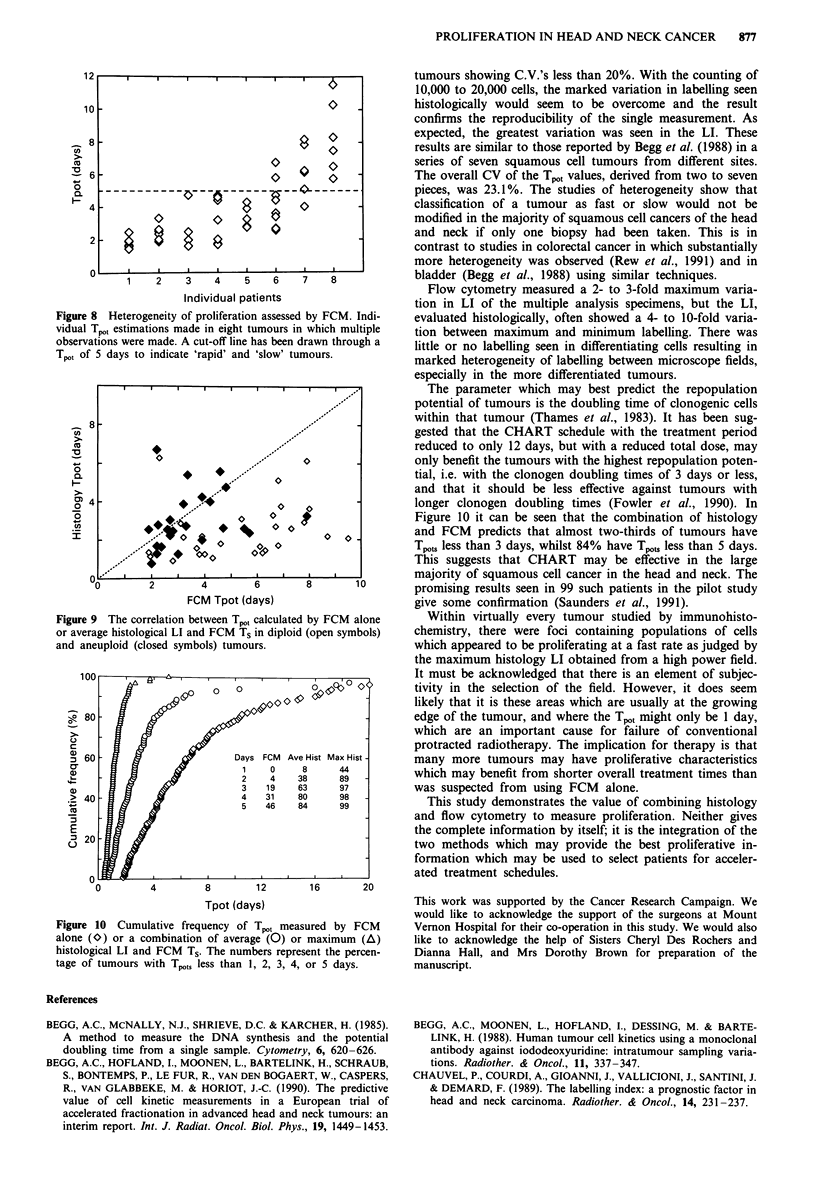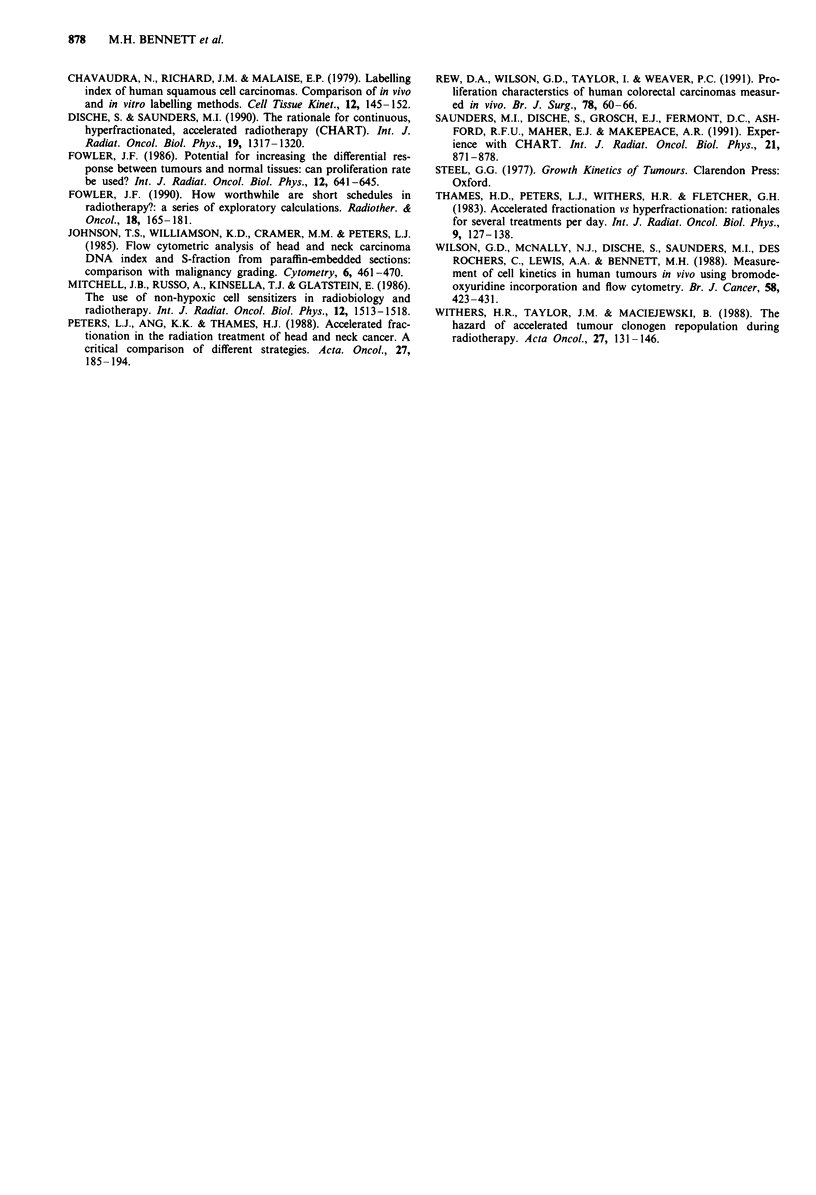# Tumour proliferation assessed by combined histological and flow cytometric analysis: implications for therapy in squamous cell carcinoma in the head and neck.

**DOI:** 10.1038/bjc.1992.183

**Published:** 1992-06

**Authors:** M. H. Bennett, G. D. Wilson, S. Dische, M. I. Saunders, C. A. Martindale, B. M. Robinson, A. E. O'Halloran, M. D. Leslie, J. H. Laing

**Affiliations:** Histopathology Department, Mount Vernon Hospital, Northwood, UK.

## Abstract

**Images:**


Br. J. Cancer (1992), 65, 870-878 © Macmillan Press Ltd., 1992

### Tumour proliferation assessed by combined histological and flow cytometric analysis: implications for therapy in squamous cell carcinoma in the head and neck

M.H. Bennett[1], G.D. Wilson[2], S. Dische[3], M.I. Saunders[3], C.A. Martindale[2], B.M. Robinson[1], A.E. O'Halloran[1], M.D. Leslie[3] & J.H.E. Laing[4]

[1]*Histopathology Department,* [2]*CRC Gray Laboratory,* [3]*Marie Curie Research Wing, and* [4]*RAFT Department of Research in Plastic Surgery, Mount Vernon Hospital, Northwood, UK.*

**Summary** The two techniques of flow cytometry analysis (FCM) and immunohistochemical localisation of bromodeoxyuridine (BrdUrd) incorporation after *in vivo* administration, were combined to study proliferation in squamous cell carcinoma of the head and neck region. Care was taken in this study to ensure that similar material was processed using both techniques such that comparisons could be made. FCM underestimated the labelling index (LI) in tumours classified as diploid compared to the histological evaluation of the tumour cells within those tumours (4.6% *vs* 17.1.%). However, in aneuploid tumours, the FCM LI (10.7%) was similar to that obtained from histology (13.5%). Indeed, proliferation assessed by the combination of histology LI and FCM duration of S-phase ($T_S$) indicated that diploid tumours had a shorter median potential doubling time ($T_{pot}$) of 2.1 days compared to aneuploid (2.8 days). Despite the heterogeneity of proliferation evident histologically within the specimens, there was not a wide variation in the results of FCM analysis when multiple samples from resections were studied. Using FCM data alone, 46% of the tumours showed a $T_{pot}$ of less than 5 days. When the $T_S$ from the FCM data was combined with the average histological LI, 84% were less than 5 days and with the maximum LI, 99% were within this time interval. Compared with previous estimates, the proportion of tumours possessing proliferative characteristics which may indicate the need for acceleration of treatment seems to be much larger.

Persistent or recurrent tumours remains a problem for many patients with advanced cancer of the head and neck. Recent evidence derived from the cellular kinetics of human tumours and from analyses of clinical data has suggested that an important cause for radiation failure may be cellular repopulation during the course of treatment (Fowler, 1986; Withers *et al.*, 1988; Wilson *et al.*, 1988).

Accelerated schedules of radiotherapy have been devised which reduce the overall duration of treatment so as to minimise the time for cellular repopulation (Peters *et al.*, 1988; Dische & Saunders, 1990). However, some reduction in the total dose given to tumour may be necessary because of an increase in acute normal tissue reactions. Although those patients with tumours having a potential for rapid cellular proliferation may benefit from the accelerated regime of radiotherapy, those with slowly proliferating tumours may not and, because the total dose given may be reduced, they may be at disadvantage (Thames *et al.*, 1983). A considerable effort, therefore, has been directed to the prediction of tumours likely to benefit from accelerated radiotherapy.

Attention has focused upon the cell kinetics of human tumours and their possible prognostic significance (Wilson *et al.*, 1988; Begg *et al.*, 1990). The growth of a tumour depends upon three main parameters – the proportion of proliferating cells (the growth fraction), the duration of the cell cycle ($T_C$) and the proportion of cells which fail to take part in further cell devision (the cell loss factor). Steel (1977) introduced the potential doubling time ($T_{pot}$), as a measure of the theoretical proliferative capability of a tumour cell population in the absence of any cell loss. It seems probable that early in a course of radiotherapy, and also during chemotherapy, when many tumour cells will have been killed, cell loss factors are greatly altered and the potential cell doubling time of the tumour may be realised.

Until recently, it has not been possible to measure easily these parameters in human tumours because the determination of cell kinetics has required the administration of radioactive DNA precursors such as tritiated thymidine ($^3$HTdR) with the need for multiple biopsies to construct a pulse label mitosis analysis; in addition, many weeks are required for the result to be obtained. Simple determination of the LI can be performed by *in vitro* techniques using a variety of methods, but all calculations derived from them need to assume a cell cycle time.

Using bromodeoxyuridine (BrdUrd) and a technique developed by Begg *et al.* (1985), it is now possible to determine tumour cell kinetics in human tumours simply, safely and speedily. Using flow cytometry, the duration of S-phase ($T_S$) and the labelling index (LI) can be measured from a single sample and from these parameters, the $T_{pot}$ is calculated. Since 1985, we have been giving patients an intravenous injection of BrdUrd 4 to 6 h prior to biopsy or surgical removal and the series now extends to over 500 patients.

The administration of BrdUrd *in vivo* represents the best mode to study DNA precursor incorporation as it is free from the artifacts which can be associated with *in vitro* incorporation. However, there are possibilities of variation even *in vivo* because of poor diffusion and transient alteration in the vascular perfusion.

Using flow cytometry, however, the prerequisite for a single cell suspension loses information concerning spatial heterogeneity and cellular identity, particularly in diploid tumours where tumour cells cannot be differentiated from normal cells. Using immunohistochemical techniques, it is possible to visualise and estimate the BrdUrd labelling in tumour cells in histological sections and to compare it with that determined by flow cytometry. This paper reports the finding by flow cytometry and by histological study after BrdUrd administration, and discusses the implications of the results for clinical radiotherapy.

### Materials and methods

### *Case selection*

Commencing in 1985, all patients with head and neck tumours receiving radiotherapy in the Cancer Treatment

Correspondence: J.D. Wilson.
Received 6 December 1991; and in revised form 28 February 1992.

Centre or surgery in the Plastic Surgery Centre at Mount Vernon Hospital were considered for tumour cell kinetic studies. This was performed whenever possible and always with the patients' informed consent. Accessible tumours within the oral cavity and oropharynx were usually sampled under local anaesthesia, as also were secondary nodes in the neck using a drill biopsy or Trucut needle. In other cases, an opportunity for study often occurred at the time of an examination under anaesthesia or at resection of tumour.

The dose of BrdUrd administered to patients, in this study, was 200 mg by intravenous, bolus injection in 20 ml of normal saline. The desired interval between administration of BrdUrd and the taking of the tumour sample was 4 to 6 h, although in some cases, for practical reasons, longer intervals elapsed.

The majority of biopsies under local anaesthesia were performed by one operator (S.D.) and a consistent policy of division of the sample was followed to ensure that the pieces taken for histology and FCM were macroscopically similar. A small portion was placed in formal saline for histological study and the rest into 70% ethanol for cytokinetics. Where a whole tumour was excised, the specimen was taken to the Department of Histopathology, cut immediately by the pathologist and appropriate adjacent samples taken. Clinical data was recorded and follow-up performed in all cases.

Out of a total of 502 patients studied with BrdUrd between May 1985 and June 1991, 162 were in patients with squamous cell cancers in the head and neck region. Of these, 123 cases are presented in which both FCM and immunohistochemical labelling have been assessed.

*Flow cytometry*

The flow cytometry methods of preparation and analysis have been described in detail elsewhere (Wilson *et al.*, 1988). Briefly, ethanol-fixed tissue fragments were digested into nuclei using 0.4 mg ml$^{-1}$ pepsin in 0.1 mol l$^{-1}$ HCl for 30 min at 37°C. DNA was denatured with 2 mol l$^{-1}$ HCl for 12 min at room temperature. A 1:20 dilution of a rat anti-BrdUrd antibody (Sera Labs, Crawley Down) in PBS containing 0.5% Tween-20 and 0.5% normal goat serum was incubated with the nuclei for 1 h at room temperature. Fluorescence was indirectly attached to the BrdUrd using a 1:20 dilution of goat anti-rat IgG FITC conjugate (Sigma Chemical Co., Poole) for 30 min at room temperature. Total DNA was stained using 10 $\mu$g ml$^{-1}$ propidium iodide and the samples analysed by FCM.

The majority of specimens were analysed on an Ortho Systems 50-H Cytofluorograf, although some were analysed using a Becton Dickinson FACScan. Both machines were equipped with pulse processing facilities to discriminate cell doublets. At least 10,000 events were collected in list mode.

The data derived from the FCM profiles were the DNA index, the LI of all cells or the aneuploid subcomponent in appropriate tumours making a simple correction for cell division (Wilson *et al.*, 1988), the $T_S$ using the method of Begg *et al.* (1985) and the $T_{pot}$ using the formula

$$T_{pot} = \lambda . \frac{T_S}{LI}$$

where $\lambda$ was assumed to be 0.8.

*Histology*

Specimens for histological examination were fixed in formal saline and processed to paraffin wax blocks, 5 $\mu$m sections cut in the standard manner and mounted on poly-l-lysine coated slides. One section was stained with haematoxylin and eosin and from this the type of tumour and its grade were determined, and also the proportions of tumour, normal non-neoplastic tissues and necrosis or debris estimated. An adjacent section was immuno-stained for BrdUrd using the Avidin Biotin Complex (ABC) technique. Pretreatment with 0.1% trypsin in 0.1% calcium chloride for 13 min at 37°C, followed by incubation in 1 mol l$^{-1}$ HCl for 15 min at 37°C was required to expose the binding sites for BrdUrd. The monoclonal antibody used in these studies was mouse anti-BrdUrd (Dakopatts, High Wycombe, England) at 1:30 in Tris buffered saline containing 1% human AB serum. The monoclonal antibody against BrdUrd was different to that used in the FCM studies, as mouse determinants are routinely used in the ABC method.

All stained sections were quantitated by assessment of the whole specimen using a visual estimate, whilst 15 (about 10%) were also counted using a graticule for comparison. The visual technique involved selecting low power areas of lowest and highest labelling of the tumour cells. One high power field ( $\times$ 40 objective $\times$ 10 eyepiece) of each of these areas was counted using a video system with a grid on the screen or with a square graticule filling half the diameter of the field. In both methods, the field contained between 50 and 200 tumour cells, depending on their size and density. The specimen was then carefully scanned and an estimate of average tumour cell LI made based on the maximum and minimum LI's and the distribution of these throughout the specimen. In addition, the pattern of staining, which gave an indication of heterogeneity, was also assessed (see Figure 2).

In 15 cases, labelling was assessed by point counting using alternate high power fields ( $\times$ 40 objective $\times$ 10 eyepiece) and a square graticule. The field contained 50 to 350 tumour cells, such that 10 to 20 high power fields were counted for each specimen to obtain a minimum of 2,000 cells. The average LI was calculated as the total number of BrdUrd labelled cells *vs* the total tumour cells counted. Standard errors were computed from the variation between high power fields.

There was considerable heterogeneity in the intensity of BrdUrd labelling both within and between specimens. However, the discrimination of even lightly labelled cells from those which were not labelled was straightforward due to manipulation of the haematoxylin staining.

The histology LI does not take into account cell division between injection and biospy. A correction for this was made using the FCM data in which the fraction of BrdUrd labelled cells which have either divided or remain undivided is known. The histology LI was corrected by a simple proportion from the uncorrected and corrected FCM LI by:

$$\text{corrected LI (hist)} = \frac{\text{corrected LI (FCM)}}{\text{uncorrected LI (FCM)}} \times \text{uncorrected LI (hist)}$$

### Results

*Comparison of point counting and visual estimation of histological LI*

Figure 1 shows the correlation between high power field counting and the visual estimate of LI on tissue sections. There was a highly significant correlation ($r^2 = 0.93$, $P < 0.0001$) with a slope of 0.91.

*Data obtained from histology and FCM*

Figure 2 shows examples of staining profiles obtained from immunohistochemistry. Four different staining patterns were observed and these were denoted marginal, intermediate, random and mixed, i.e. when more than one of the other patterns were observed. Marginal staining was most commonly found in grade 1 tumours and random in grade 4, with intermediate patterns in grades 2 and 3. Four examples have been chosen to illustrate different staining patterns. Figure 2a shows a grade 1 tumour of the tongue showing marginal staining when only the cells at the margin of the tumour cords and masses were labelled. In this specimen, the corrected average LI was 17.4%. This was in contrast to the FCM data, in which the LI was only 3.9% (Figure 3a). The tumour was diploid and had a $T_S$ of 9.1 h, resulting in a $T_{pot}$ of 7.8 days. The discrepancy in LI may be explained by the histological observation that only 20% of the specimen was occupied by tumour cells, the other 80% being stromal cells.

**Figure 1** Correlation between point counting and the visual estimate method for the assessment of BrdUrd LI on histological sections. The data represent the observed value for the visual method and the overall mean value for point counting. The bars represent s.e.m.'s calculated from all the high power fields. No correction for cell division has been made.

Figure 2b shows an example of an intermediate staining pattern in which several layers of cells around the margin are labelled. This was a grade 3 tumour of the upper alveolus which had an average LI of 13.4%. This LI agreed well with that from FCM (Figure 3b), 11.8%, as the tumour was aneuploid with a DNA index of 1.85. The $T_S$ and $T_{pot}$ were 9.9 h and 2.8 days respectively.

The other distinct pattern, random, is shown in Figure 2d, in which labelled cells were scattered throughout. The example shows a grade 4 tumour of the lower alveolus in which the histology LI was 28%, whilst FCM (Figure 3d) gave a value of 17.2%. The LI and $T_S$ of 10.3 h combined to give a rapid $T_{pot}$ of only 2.0 days.

The mixed patterns usually correlated with tumours showing areas of differing degrees of differentiation, but the grade given with each tumour was for the most poorly differentiated area. Figure 2c shows a grade 3 tumour of the tonuge with a LI of 20.1%. In this tumour some areas showed moderate differentiation with an intermediate labelling pattern, although many layers of cells in the periphery of the cell mass were labelled. Other less differentiated areas (arrowed) show a random labelling pattern. FCM (Figure 3c) revealed an aneuploid tumour (DNA index 1.85) with a LI, $T_S$ and $T_{pot}$ of 16.8%, 13.4 h and 2.7 days respectively.

*Distribution of proliferation parameters*

Table I shows the median values obtained for the parameters studied for each group of tumours. There are no important differences in proliferation characteristic between primary, recurrent or metastatic disease, although the numbers in some groups are small.

Within each of the parameters measured there was a wide distribution of values as indicated by the numbers in parentheses in Table I (the coefficients of variations for all tumours) and from Figure 4. Figure 4 shows the distribution of FCM LI, $T_S$, $T_{pot}$ and average histological LI in primary tumours only. Flow cytometry derived LI varied from 0.9 to 20.4%, whilst that obtained from histology ranged from 1.8 to 45.2%. The variation in LI was expected but $T_S$ also showed a wide variation ranging from 5.4 to 24.3 h. The two FCM parameters yield the $T_{pot}$ which can be from as short as 1.8 days to 41.2 days. This parameter shows most variation as judged by the C.V. of 82%.

*Influence of site on proliferation parameters*

The major sites studied in the head and neck classified according to grade, DNA index, $T_{pot}$ and average LI assessed histologically, are shown in Figure 5. When the sites are compared in order from lip to tonsil, there is a trend for the proportion of low grade tumours to decrease and for the incidence of aneuploidy to increase. At the columella, there was also a high incidence of aneuploidy. There was no significant relationship between proliferation characteristics and site. Tumours of the columella and floor of mouth tended to show the slowest proliferation when assessed flow cytometrically, but this discrimination was not apparent when histology was used to calculate LI. Both methods agreed that tumours arising in the tonsil region tended to be rapidly proliferating.

*Relationship between proliferation, histological grading and clinical staging*

Figure 6 relates grade and T staging to proliferation. None of the proliferation parameters, FCM LI, $T_S$, $T_{pot}$, average LI or maximum LI showed any significant relationship with histopathological grading. Interestingly, grade 4 tumours tended to be diploid and they were inclined to be more slowly proliferating than grades 2 and 3 when assessed by FCM. However, histologically, the proportion of BrdUrd labelled cells was higher in grade 4 tumours in which the median histology LI was 22.4% compared to 11.8, 15.7 and 13.9 in grades 1, 2 and 3 respectively.

Clinical staging also had little relationship to proliferative characteristics. Only two patients presented $T_1$ tumours and the median $T_{pots}$ for $T_2$, $T_3$ and $T_4$ were 4.8, 5.9 and 4.6 days respectively. Histological evaluation of LI suggested that the tumour cell LI may be lower in $T_2$ tumours, in which the median values was 9.6%, compared to 16.4 and 16.5% in stages 3 and 4. There were no apparent relationships between N stage and proliferative characteristics, median $T_{pots}$ were 6.0, 4.8, 4.9 and 5.4 days, in $N_0$, $N_1$, $N_2$ and $N_3$ respectively and the histology LI's were 13.7, 16.7, 16.3 and 12.7% within these categories.

*Influence of DNA index on proliferation*

As expected, FCM analysis suggested that diploid tumours have a lower LI than aneuploid tumours (median values 4.6 and 10.7% respectively) (Figure 7). However, this difference did not exist when in histological evaluation only the tumour cells were assessed. In fact, the average histological LI was slightly greater in diploid (17.1%) than aneuploid tumours (13.5%), where the value was of the same order as that for LI by FCM (10.7%). $T_S$ was slightly shorter in diploid (9.0 h) than aneuploid tumours (11.7 h). When the $T_{pot}$ was calculated from the FCM data, diploid tumours appear to be more slowly proliferating (6.8 days) than aneuploid (3.9 days). However, if the average histological LI was used in conjunction with FCM $T_S$ for each tumour, the median $T_{pot}$ in diploid tumours was only 2.1 days and was faster than that found in aneuploid tumours (2.8 days).

*Heterogeneity of proliferation assessed by FCM*

Heterogeneity was assessed in eight patients, whose tumours were resected, by taking 4 and 9 (mean 6) individual biopsy-like fragments from the tumours and staining and assessing each one by flow cytometry.

As expected, each proliferation parameter showed variation as judged by the coefficient of variation (CV). However, the variations were not large. The average CV's for LI, $T_S$ and $T_{pot}$ were 26.8%, 15.1% and 30.3% respectively (c.f. Table I). The individual data for the $T_{pots}$ are shown in Figure 8. The mean $T_{pots}$ spanned from 1.85 days to 8.32 days. It is apparent that few tumours would be wrongly classified as fast or slow (in this case above or below a $T_{pot}$ of 5 days) unless their mean value was close to the cut-off value.

There was evidence of DNA index variation in only two of these eight tumours. In two tumours, all observations were diploid, in a further four, all observations were aneuploid with similar DNA indices within each tumour. Two tumours

**Figure 2** Immunohistochemical localisation of BrdUrd in four squamous cell cancers of the head and neck. The H and E and immunoperoxidase staining patterns from adjacent sections are shown for a tumour showing **a**, a marginal (magnification × 140), **b**, intermediate (magnification × 400), **c**, mixed (magnification × 100), and **d**, random staining pattern (magnification × 170). For explanation of the staining patterns, see text.

**Figure 3** Corresponding flow cytometric distributions of BrdUrd and total DNA content in the four tumours presented in Figure 1.

showed two or more DNA indices. In one of these, five of six pieces were diploid and one was tetraploid. In the other tumour, one piece was diploid, one was tetraploid, one had a DNA index of 1.9 and the remaining three were close to 1.8.

### Discussion

The incorporation of BrdUrd into human tumours *in vivo* has facilitated the study of cell kinetics. Potential cell doubling times can be estimated from a single biopsy taken several hours after the injection of BrdUrd using flow cytometric techniques. However, the major drawback of FCM is the loss of morphological cellular identity and the possibility that cell selection may occur. These limitations present a particular problem in tumours classified as diploid by FCM, in which discrimination of normal and tumour cells is not possible. In this study using immunohistochemical localisation of proliferating cells in histological sections of adjacent comparable specimens, we were able to identify the cellular composition of the tissue examined.

The choice of the visual method to assess LI on tissue sections was made because point counting of 10 to 20 high power fields to achieve a total count of 2,000 cells may only examine a fraction of the whole specimen. It will also be highly dependent on the choice of fields if there is variability and structure to the labelling patterns, as is found in the better differentiated squamous cell cancers. The visual method, like FCM, studies the whole specimen. The technique involves quantitation of maximum and minimum labelling areas and a careful assessment of the distribution of labelling throughout the whole specimen. The correlation in Figure 1, between point counting and the visual estimate, justifies the approach used in this study. However, it should be recognised that this method would not be suitable for counting mitoses where the mitotic index may be less than 1%.

The addition of histological evaluation of proliferating cells to the results of FCM in squamous cell cancer of the head and neck reveals that there is no systematic difference in proliferation between diploid and aneuploid tumours (Figure 7). The average LI of diploid tumours was 17.1% and of aneuploid tumours 13.5%. The $T_{pot}$ was calculated using the individual histology derived LI and the flow cytometry derived $T_S$. Figure 9 shows the correlation between the hybridrised $T_{pot}$ and that derived purely from FCM. There is good agreement in aneuploid tumours between two approaches. This result suggests that cell selection is not a problem with the FCM technique, but proliferation in diploid tumours is underestimated by FCM alone. This calls into question the validity of conclusions drawn from kinetic studies, particularly those where data is available only from DNA S-phase fractions, which suggest that diploid tumours are more slowly proliferating (Johnson *et al.*, 1985). Clearly this is not the case for head and neck tumours, and we have seen, in preliminary review, that neither is it so in oesophageal or lung carcinoma. Because diploid tumours tended to have a shorter $T_S$ than aneuploid tumours (a measurement which should not be greatly compromised by differences in the detection of labelled cells by FCM), the median $T_{pot}$, determined using average histological LI, was shorter in diploid tumours at 2.1 days compared to 2.8 days for the aneuploid. The finding that diploid tumours are not more slowly proliferating than aneuploid tumours may seem surprising. However, there is no direct evidence in the literature to suggest that this may not be the case; studies involving $^3$HTdR LI have not incorporated FCM analysis of DNA content, and DNA S-phase fraction analysis alone is not appropriate for the reasons outlined in this manuscript. Indirect evidence comes from the difference in response of diploid or aneuploid tumours to radio- or chemotherapy, but any difference cannot be attributed solely to a difference in proliferation.

There was a trend for low grade tumours and the lower LI's to be found at the front of the mouth compared to those originating further back, but our results did not not suggest any strong relationship between the site of the tumour within the head and neck and proliferation assessed either by FCM or by histology. These results are in agreement with Chauvel *et al.* (1989) using $^3$HTdR. In our study, and that of Chauvel *et al.* (1989) and Johnson *et al.* (1985), there was no relationship between grading and proliferation.

The choice of technique to assess cell kinetics will have profound influence on the proliferative classification of individual tumours. In this study, the ranking of patients differs if FCM is used alone compared to FCM and histology,

**Table I** Median values for each proliferation parameter in primary and recurrent head and neck cancer. The numbers in parentheses represent the coefficient of variation for each parameter for all tumours

| Group | No. | *LI (%)* | *$T_S$ (h)* | *$T_{pot}$ (days)* | *Ave LI (%)* | *Max LI (%)* | *% Aneuploid* |
|---|---|---|---|---|---|---|---|
| All | 123 | 6.8 (66) | 9.9 (33) | 5.7 (82) | 14.9 (58) | 33.9 (40) | 41 |
| Untreated | | | | | | | |
| primary | 91 | 6.9 | 9.7 | 5.2 | 14.5 | 33.8 | 41 |
| neck nodes | 9 | 5.4 | 11.4 | 7.7 | 20.9 | 27.0 | 33 |
| Recurrent | | | | | | | |
| primary | 13 | 6.8 | 12.3 | 6.1 | 9.4 | 36.0 | 46 |
| neck nodes | 3 | 6.2 | 11.1 | 3.7 | 6.0 | 22.2 | 66 |
| distant metastases | 7 | 4.8 | 9.9 | 6.3 | 17.6 | 43.1 | 43 |

Figure 4 Distribution of proliferation parameters in primary cancer of the head and neck.

Figure 5 Influence of tumour site. The distribution of histological grading and DNA aneuploidy, and median $T_{pot}$ and average histological LI, presented for each of the major sites for primary tumours studied.

mainly due to difference in classification of diploid tumours. However, if histological LI is used alone, which would be analogous to studies employing *in vitro* $^3$HTdR incorporation and autoradiography, there is also a difference in ranking compared to the combination of FCM and histology. This is because $T_S$ shows wide variation in this and every other group of human tumours we have studied. Figure 4 shows that $T_S$ can be as short as 5.4 h and as long as 24.3 h in head and neck tumours. In addition, diploid tumours tended to have a shorter $T_S$ than aneuploid tumours. *In vitro* labelling with BrdUrd or $^3$HTdR is inadequate to fully characterise the cellular kinetics of solid tumours.

The intravenous administration of BrdUrd to patients for tumour cell kinetic study has been questioned as a safe procedure. It is a cytotoxic drug which remains under study as a radiosensitiser (Mitchell *et al.*, 1986). The dose of

**Figure 6** The relationship between proliferation and histological grading or T staging. The individual data is presented for each tumour. The lines on each graft represent the median value for each proliferation parameter and each particular grade or stage.

**Figure 7** The influence of DNA aneuploidy on proliferation parameters. The data represent median values for each parameter for diploid and aneuploid tumours. ■, Diploid; □, Aneuploid.

200 mg currently employed for this diagnostic procedure can be compared with 7–10 gram commonly used with satisfactory tolerance therapeutically. In the administration of BrdUrd, in current dosage, to over 500 patients, no immediate or late effect has been observed.

The uptake of BrdUrd in tumour cells after *in vivo* administration will depend upon drug distribution, diffusion and upon the integrity of the vascular supply within the tumour. *In vitro* labelling is also subject to diffusion problems, but more importantly upon the viability of cells in isolated tissue. The quantitation of *in vitro* labelled tissue fragments is also more subjective as often only peripheral areas can be scored. However, the only study which has compared *in vitro* and *in vivo* labelling in squamous cell carcinomas of the head and neck, using $^3$HTdR, found no systematic difference between the two methods in the estimation of LI (Chavaudra *et al.*, 1979). If anything, the *in vitro* method slightly underestimated the LI. In addition, their median LI's (17.0% *in vivo*, 11.5% *in vitro*) were similar to that obtained in this present study using BrdUrd and histology (14.9%).

Heterogeneity is a valid criticism levelled at techniques which rely on biopsy material particularly, as in the case of the FCM method, when a temporal measurement ($T_S$) is being made from one observation. It was not possible to study heterogeneity using FCM in more than a small proportion of our cases, as much of the material was obtained by biopsy. However, in the eight specimens where multiple samples were studied, heterogeneity of the FCM-derived proliferation parameters was surprisingly small. The $T_S$ in particular proved to be the least variable, with six of the eight

**Figure 8** Heterogeneity of proliferation assessed by FCM. Individual $T_{pot}$ estimations made in eight tumours in which multiple observations were made. A cut-off line has been drawn through a $T_{pot}$ of 5 days to indicate 'rapid' and 'slow' tumours.

**Figure 9** The correlation between $T_{pot}$ calculated by FCM alone or average histological LI and FCM $T_S$ in diploid (open symbols) and aneuploid (closed symbols) tumours.

**Figure 10** Cumulative frequency of $T_{pot}$ measured by FCM alone (◇) or a combination of average (○) or maximum (△) histological LI and FCM $T_S$. The numbers represent the percentage of tumours with $T_{pots}$ less than 1, 2, 3, 4, or 5 days.

tumours showing C.V.'s less than 20%. With the counting of 10,000 to 20,000 cells, the marked variation in labelling seen histologically would seem to be overcome and the result confirms the reproducibility of the single measurement. As expected, the greatest variation was seen in the LI. These results are similar to those reported by Begg *et al.* (1988) in a series of seven squamous cell tumours from different sites. The overall CV of the $T_{pot}$ values, derived from two to seven pieces, was 23.1%. The studies of heterogeneity show that classification of a tumour as fast or slow would not be modified in the majority of squamous cell cancers of the head and neck if only one biopsy had been taken. This is in contrast to studies in colorectal cancer in which substantially more heterogeneity was observed (Rew *et al.*, 1991) and in bladder (Begg *et al.*, 1988) using similar techniques.

Flow cytometry measured a 2- to 3-fold maximum variation in LI of the multiple analysis specimens, but the LI, evaluated histologically, often showed a 4- to 10-fold variation between maximum and minimum labelling. There was little or no labelling seen in differentiating cells resulting in marked heterogeneity of labelling between microscope fields, especially in the more differentiated tumours.

The parameter which may best predict the repopulation potential of tumours is the doubling time of clonogenic cells within that tumour (Thames *et al.*, 1983). It has been suggested that the CHART schedule with the treatment period reduced to only 12 days, but with a reduced total dose, may only benefit the tumours with the highest repopulation potential, i.e. with the clonogen doubling times of 3 days or less, and that it should be less effective against tumours with longer clonogen doubling times (Fowler *et al.*, 1990). In Figure 10 it can be seen that the combination of histology and FCM predicts that almost two-thirds of tumours have $T_{pots}$ less than 3 days, whilst 84% have $T_{pots}$ less than 5 days. This suggests that CHART may be effective in the large majority of squamous cell cancer in the head and neck. The promising results seen in 99 such patients in the pilot study give some confirmation (Saunders *et al.*, 1991).

Within virtually every tumour studied by immunohistochemistry, there were foci containing populations of cells which appeared to be proliferating at a fast rate as judged by the maximum histology LI obtained from a high power field. It must be acknowledged that there is an element of subjectivity in the selection of the field. However, it does seem likely that it is these areas which are usually at the growing edge of the tumour, and where the $T_{pot}$ might only be 1 day, which are an important cause for failure of conventional protracted radiotherapy. The implication for therapy is that many more tumours may have proliferative characteristics which may benefit from shorter overall treatment times than was suspected from using FCM alone.

This study demonstrates the value of combining histology and flow cytometry to measure proliferation. Neither gives the complete information by itself; it is the integration of the two methods which may provide the best proliferative information which may be used to select patients for accelerated treatment schedules.

This work was supported by the Cancer Research Campaign. We would like to acknowledge the support of the surgeons at Mount Vernon Hospital for their co-operation in this study. We would also like to acknowledge the help of Sisters Cheryl Des Rochers and Dianna Hall, and Mrs Dorothy Brown for preparation of the manuscript.

### References

BEGG, A.C., MCNALLY, N.J., SHRIEVE, D.C. & KARCHER, H. (1985). A method to measure the DNA synthesis and the potential doubling time from a single sample. *Cytometry*, **6**, 620–626.

BEGG, A.C., HOFLAND, I., MOONEN, L., BARTELINK, H., SCHRAUB, S., BONTEMPS, P., LE FUR, R., VAN DEN BOGAERT, W., CASPERS, R., VAN GLABBEKE, M. & HORIOT, J.-C. (1990). The predictive value of cell kinetic measurements in a European trial of accelerated fractionation in advanced head and neck tumours: an interim report. *Int. J. Radiat. Oncol. Biol. Phys.*, **19**, 1449–1453.

BEGG, A.C., MOONEN, L., HOFLAND, I., DESSING, M. & BARTELINK, H. (1988). Human tumour cell kinetics using a monoclonal antibody against iododeoxyuridine: intratumour sampling variations. *Radiother. & Oncol.*, **11**, 337–347.

CHAUVEL, P., COURDI, A., GIOANNI, J., VALLICIONI, J., SANTINI, J. & DEMARD, F. (1989). The labelling index: a prognostic factor in head and neck carcinoma. *Radiother. & Oncol.*, **14**, 231–237.

CHAVAUDRA, N., RICHARD, J.M. & MALAISE, E.P. (1979). Labelling index of human squamous cell carcinomas. Comparison of *in vivo* and *in vitro* labelling methods. *Cell Tissue Kinet.*, **12**, 145-152.

DISCHE, S. & SAUNDERS, M.I. (1990). The rationale for continuous, hyperfractionated, accelerated radiotherapy (CHART). *Int. J. Radiat. Oncol. Biol. Phys.*, **19**, 1317-1320.

FOWLER, J.F. (1986). Potential for increasing the differential response between tumours and normal tissues: can proliferation rate be used? *Int. J. Radiat. Oncol. Biol. Phys.*, **12**, 641-645.

FOWLER, J.F. (1990). How worthwhile are short schedules in radiotherapy?: a series of exploratory calculations. *Radiother. & Oncol.*, **18**, 165-181.

JOHNSON, T.S., WILLIAMSON, K.D., CRAMER, M.M. & PETERS, L.J. (1985). Flow cytometric analysis of head and neck carcinoma DNA index and S-fraction from paraffin-embedded sections: comparison with malignancy grading. *Cytometry*, **6**, 461-470.

MITCHELL, J.B., RUSSO, A., KINSELLA, T.J. & GLATSTEIN, E. (1986). The use of non-hypoxic cell sensitizers in radiobiology and radiotherapy. *Int. J. Radiat. Oncol. Biol. Phys.*, **12**, 1513-1518.

PETERS, L.J., ANG, K.K. & THAMES, H.J. (1988). Accelerated fractionation in the radiation treatment of head and neck cancer. A critical comparison of different strategies. *Acta. Oncol.*, **27**, 185-194.

REW, D.A., WILSON, G.D., TAYLOR, I. & WEAVER, P.C. (1991). Proliferation characterstics of human colorectal carcinomas measured *in vivo*. *Br. J. Surg.*, **78**, 60-66.

SAUNDERS, M.I., DISCHE, S., GROSCH, E.J., FERMONT, D.C., ASHFORD, R.F.U., MAHER, E.J. & MAKEPEACE, A.R. (1991). Experience with CHART. *Int. J. Radiat. Oncol. Biol. Phys.*, **21**, 871-878.

STEEL, G.G. (1977). *Growth Kinetics of Tumours*. Clarendon Press: Oxford.

THAMES, H.D., PETERS, L.J., WITHERS, H.R. & FLETCHER, G.H. (1983). Accelerated fractionation *vs* hyperfractionation: rationales for several treatments per day. *Int. J. Radiat. Oncol. Biol. Phys.*, **9**, 127-138.

WILSON, G.D., MCNALLY, N.J., DISCHE, S., SAUNDERS, M.I., DES ROCHERS, C., LEWIS, A.A. & BENNETT, M.H. (1988). Measurement of cell kinetics in human tumours *in vivo* using bromodeoxyuridine incorporation and flow cytometry. *Br. J. Cancer*, **58**, 423-431.

WITHERS, H.R., TAYLOR, J.M. & MACIEJEWSKI, B. (1988). The hazard of accelerated tumour clonogen repopulation during radiotherapy. *Acta Oncol.*, **27**, 131-146.